# Process of development of a contemporary curriculum in advanced midwifery

**DOI:** 10.4102/hsag.v23i0.1037

**Published:** 2018-07-31

**Authors:** Carin Maree, Mariatha Yazbek, Ronell Leech

**Affiliations:** 1 Department of Nursing Science, University of Pretoria, South Africa

## Abstract

**Background:**

We identified the need for a contemporary curriculum to enhance education in advanced midwifery. Midwifery education needs to address the changing health needs, meet the requirements of the educational framework in South Africa and align with international trends.

**Aim:**

The aim was to describe the development of a contemporary curriculum for advanced midwifery.

**Setting:**

The curriculum development took place at a South African university.

**Method:**

We used a situational analysis to create a contemporary curriculum based on the Research Development and Diffusion Model.

**Results:**

We described the process followed for the situation analysis towards the development of a contemporary curriculum in advanced midwifery which is aligned with global trends.

**Conclusion:**

A situation analysis of the existing curriculum, the community and country’s maternal and neonatal needs, educational framework and global trends should be used to develop the intended contemporary curriculum.

## Introduction

There is a demand for advanced midwives to address maternal and child mortality in South Africa while meeting the requirements of the re-engineered primary health care system amidst staff shortages (Oosthuizen & Ehlers 2007). Midwives should be competent and stay up to date with international midwifery practices (South African Nursing Council 2013).

Successful education and facilitation of learning in advanced midwifery depends on curricula that are transparent and effective in terms of goals, learning activities and assessment of learning outcomes (Nulty 2012). Curriculum structure should be continuously updated, enabling the underlying potential of students to be realised without compromising the quality of the qualification. Curricula should develop professionals who can successfully engage in today’s complex world (Council on Higher Education Report 2013). The development process of contemporary curricula has generic importance and can be used by various institutions in different contexts.

### Setting

#### Key focus

We identified the need to revise the existing curriculum in advanced midwifery at a South African university. The development of a curriculum that is current and relevant to the needs of the community and country (Democratic Nurses’ Organisation of South Africa 2013), which complies with the requirements of the educational framework (Council on Higher Education Report 2013) and is aligned with international trends (Alliance of African Midwives 2011; International Confederation of Midwives [ICM] 2012), is described in this publication.

### Background

Midwifery had developed into a highly specialised field across the globe (Alliance of African Midwives 2011; Barger 2005). Midwives who successfully complete a midwifery education programme are registered and legally licensed to practice midwifery, whereas advanced midwives are prepared as specialists in midwifery (International Council of Nurses [ICN] 2009; South African Nursing Council 2013).

Midwives and advanced midwives play a vital role in the well-being of mothers and children and can influence maternal and child mortality figures. Maternal and child mortality figures are important indicators of the health profile of communities and countries. Well-trained, competent midwives can reduce mortality, whereas poorly equipped midwives may lead to increases in mortality (World Health Organization [WHO] Every Newborn Action Plan [ENAP] 2013). The curricula used in basic and specialised midwifery training will determine the competency of midwives and need to be current, relevant and at an appropriate level (Democratic Nurses’ Organisation of South Africa 2013). Curriculum revision is therefore an important part of training and education (O’Neill 2010).

Curricula are commonly revised to benefit student learning (Wolf 2007) while meeting the needs of the country (WHO UNICEF 2010) and changing needs in maternal and newborn health (WHO ENAP 2013). Well-trained midwives have the potential to meet South Africa’s health care needs, especially in terms of continued population growth, high HIV and AIDS rates, and high maternal and infant death statistics (Statistics South Africa 2015). Well-trained nurse-midwives could alleviate the overworked and understaffed health care environment that practicing midwives are currently experiencing (Alliance of African Midwives 2011).

### Trends

Given these needs, our aim was to develop a contemporary curriculum for midwives (basic and advanced) following the Research, Development and Diffusion Model (Robinson 2009; Rogers 2003). The process included the following steps: a situation analysis (research), curriculum development and the diffusion of the curriculum into the education system (Anema & McCoy 2010; Robinson 2009; Rogers 2003) of which the first two steps are described.

The introduction of the Higher Education Qualifications Sub-Framework (Council on Higher Education 2014) and the shift to competency-based education (Council on Higher Education 2013; ICM 2012; ICN 2009; WHO 2010) necessitated the revision of midwifery curricula. To prepare competent midwives and advanced midwives, basic education should be evidence based and meet the needs of the health care system. It should produce graduates with essential competencies to be leaders who maintain and improve quality of health care and fill their role in the professional workforce. They should strengthen health systems to meet the needs of populations and protect the public while meeting global standards (Frenk et al. 2010; Ramasubramaniam & Grace 2015).

Kamp and Klaassen (2013) highlight the evolution of learning and teaching environments in terms of technology and accessibility of information. Modern education should accommodate students’ different styles of learning using innovation and current practices to achieve required competencies in the workplace.

Changing curricula can be a daunting task (Spronken-Smith et al. 2011). Changing the midwifery curriculum at the particular university will require institutional support and assimilation into current university policies.

### Objectives

We describe the development of a contemporary curriculum for advanced midwifery. We researched the need for new objectives (situation analysis) and developed an innovation (contemporary curriculum for advanced midwives).

### Contribution to the field

The situation analysis describing the need for a contemporary curriculum in advanced midwifery was the foundation for developing a contemporary curriculum that meets the community and country’s needs by delivering quality and competent midwifery care, meets the educational framework’s requirements and is aligned with international trends. The process might be useful to other institutions and for other professional specialities.

### Literature review

According to the ICM (2017a), a midwife is:

a person who has successfully completed a midwifery education programme that is duly recognized in the country where it is located and that is based on the ICM Essential Competencies for Basic Midwifery Practice and the framework of the ICM Global Standards for Midwifery Education; who has acquired the requisite qualifications to be registered and/or legally licensed to practice midwifery and use the title ‘midwife’; and who demonstrates competency in the practice of midwifery. (n.p.)

Goemaes et al. (2016) proposed the following definition of advanced midwives:

Advanced midwifery practice is characterized by a level of midwifery practice at which midwives use their expertise, management and clinical leadership skills to provide evidence-based, tailored care for women and their families independently and autonomously. Professional leadership and research skills are used to evaluate and improve practice, and to advance midwifery as a profession and science. (p. 36)

Advanced midwives are able to work independently and autonomously; they are clinical and professional leaders with expert knowledge and skills in the field of midwifery and have research skills. Their roles entail being clinicians, clinical and professional leaders, educators, consultants, managers, researchers and auditors in midwifery (Goemaes et al. 2016). Knowledgeable and skilful midwives and advanced midwives reduce maternal and neonatal mortality and morbidity through proper antenatal and postnatal care (Begley et al. 2012; Pattinson 2015) and encouraging breastfeeding (Goemaes et al. 2016).

Births attended by advanced midwives are associated with reduced costs, less invasive and unnecessary interventions such as caesarean births, labour induction, augmentation and regional anaesthesia and lower incidences of third- and fourth-degree perineal tears (Goemaes et al. 2016). Although the perinatal complications are comparable to those of physician attended births, perinatal care provided by advanced midwives is associated with more continuity and holistic care than care given by doctors.

Health care curricula tend to be fragmented, outdated and static, producing ill-equipped graduates (Frenk et al. 2010). Frenk et al. (2010) highlight the importance of replacing informative and formative curricula with transformative learning. Informative curricula lead to the acquisition of knowledge and skills to produce experts, formative curricula result in socialising students around values to produce professionals and transformative curricula develop leadership attributes to produce enlightened change agents. Transformative curricula represent a shift from memorising facts to decision-making based on searching, analysis and synthesis. There is a paradigm shift from seeking professional credentials to achieving core competencies for effective teamwork in health systems, from non-critical adoption of educational models to creative adaptation of global resources to address local priorities.

Effective curricula are feasible; meet the needs of the profession and context; are comprehensive (not overloaded or incomplete), flexible, learner-responsive and inter-collaborative; promote inter-professional education; provide a diverse experience; use current technology; and strengthen educational resources, with special emphasis on faculty development. These curricula promote a new professionalism based on competencies as objective criteria for the classification of health professionals and develop a common set of values around social accountability (Frenk et al. 2010; Ramasubramaniam & Grace 2015).

Curriculum development results in all the activities and planned learning experiences through which an institution achieves a particular kind of graduate. Unlike initial development, curriculum revision is an ongoing process to maintain quality education (Ramasubramaniam & Grace 2015). Ramasubramaniam and Grace (2015) suggest that curriculum revision should use a framework or design for the implementation of intended changes; review the vision, mission and goals of the institution; and develop measurable learning outcomes based on programme goals. A clear plan should follow for the inclusion of content, teaching strategies and assessment of the learning outcomes.

Curriculum development and revision should involve stakeholders to meet the needs of the community, to create collegiality between practice and education and develop a sense of ‘ownership’ of the curriculum (Keogh et al. 2010). The core curriculum should be developed through consensus as an engaged six-step process with stakeholders, using stimulus questions; brainstorming; sharing, clarification and clustering of ideas; second-phase brainstorming, clarification and clustering; prioritisation; vote tally and eventual categorisation (Hanekom, Unger & Cilliers 2014).

Core curricula should include relevant content, contribute to safe and effective practices and be evidence based (Hanekom et al. 2014). Curricula should consider international and national standards for practice and education, health statistics, the infrastructure of the country, health systems and education institutions (Ramasubramaniam & Grace 2015).

Curricula should accommodate worldwide health challenges including significant gaps, health care needs, inequities, new infections, environmental and behavioural risks, costly health care and shortages of health care providers (Frenk et al. 2010). Rapid changes are taking place in the world of education and science and should be accounted for during development and revision of curricula (Frenk et al. 2010; Keogh et al. 2010; Ramasubramaniam & Grace 2015).

## Research design

We used a qualitative research design to direct the revision of the existing curriculum. The process consisted of two steps, namely a situation analysis and development of a contemporary curriculum. We explored the process of curriculum revision extensively from a contextual perspective. Data were obtained from written texts or from interactions with stakeholders. Qualitative content analysis identified themes and sub-themes explaining the situation analysis.

### Unit of analysis and sampling

Relevant documents were analysed to inform the contemporary curriculum and included the following:

- Documentation that described the education framework: South African Nursing Council regulations and directives; regulation books of the Faculty of Health Sciences; and official documents related to the curriculum of advanced midwifery.
- Literature related to the needs and demands of midwifery practice.
- Minutes of a stakeholders’ meeting which was held with 26 key persons in midwifery from various universities, nursing colleges, policy-makers and clinical practice.
- Report on the input obtained from midwifery experts on the contemporary curriculum for advanced midwifery.

### Data collection and treatment

We used the Research, Development and Diffusion Model (Anema & McCoy 2010; Robinson 2009; Rogers 2003) as a framework to guide the development of a contemporary curriculum in advanced midwifery.

Documents were searched for evidence regarding the status of the curriculum in relation to the actual needs of the community and country. The demands placed on practicing advanced midwives were considered during curriculum development. We identified curriculum requirements of the educational framework and international trends in advanced midwifery curricula.

## Results

The development of the contemporary curriculum took place over two years and included a stakeholder meeting followed by input from experts. We identified three main themes, namely the educational framework for the contemporary curriculum, expectations regarding the curriculum and a need for international alignment.

### Educational framework

Educational framework for curriculum was based on recommendations from the professional regulatory body (South African Nursing Council) (South Africa 2005), the higher education regulatory body (Council on Higher Education) (South Africa 1997; 2007; 2008) and the relevant regulations of the educational institutions (University of X 2012).

Midwives should be on the level of a postgraduate advanced diploma. Advanced midwives should be at the level of a professional master’s degree where successful completion requires ‘a high level of theoretical engagement and intellectual independence and have the ability to relate knowledge to the resolution of complex problems in appropriate areas of professional practice’ (Council on Higher Education 2014:35).

### Expectations regarding the curriculum

The minutes (University of X 2011) of a meeting with stakeholders from practice, education and policy-making authorities was used to distinguish between competencies of midwifery and advanced midwifery. Midwives should be able to deliver babies safely and be equipped to manage emergency situations. Midwives need to manage antenatal care and be able to address pregnancy complications and refer where necessary. Midwives should be aware of the health needs of the surrounding communities and should provide holistic care aimed at family planning and HIV prevention. Advanced midwifery should incorporate all the proficiencies of midwifery with an added advanced clinical skills, research and teaching component.

Curriculum development should address the community’s health needs according to changes in health profile, contemporary issues and health priorities (Department of Health, South Africa [S.A.] 2013). Contemporary curricula should recognise the needs of students by embracing new ideologies that have a stronger focus on student-centred learning and increased use of technology and social media (WHO 2013). Technology and social media can form bridges between health care workers and surrounding communities and their use should be promoted in the learning process.

### Need for international alignment

Educational outcomes should align with the international development of maternity services such as the Midwifery 2020 vision (Jill Rogers Associates 2010) and incorporate global trends in health education (ICN 2009; WHO 2010). The Midwifery 2020 vision was released in the United Kingdom and highlights the important role that midwives can play towards addressing inequalities in health care and contribute to public health issues. Successful midwifery education will increase the profile of the profession and attract future learners.

A summarised table provides the main themes and topics included in the contemporary curriculum, which is presented as a master’s degree by means of coursework over a period of two academic years. The research component is not captured in the table. An important shift that took place was the inclusion of relevant trend-setting documents in midwifery from entities such as the WHO (2010) and the ICM (2017b).

## Ethical considerations

As no human subjects were included in the research, approval from the faculty’s research ethics committee was not required. The privacy of the stakeholders and experts are respected as no names are disclosed. The minutes of the stakeholder meeting and the report on the input obtained from midwifery experts on the contemporary curriculum for advanced midwifery, included as part of the data collection, were accessed with the permission of the Head of the Department.

### Potential benefits and hazards

As this was a study based on an analysis of documents, no hazards were foreseen. This study may potentially benefit the needs of the community and country if the contemporary midwifery curriculum is accepted and implemented by all nursing education institutions in South Africa. A contemporary curriculum will improve the quality of midwifery care.

### Recruitment procedures

We analysed documents, and hence, no participants were recruited.

### Informed consent

No participants were involved and informed consent was unnecessary.

### Data protection

Although the data were collected from documents and literature, the researchers still maintained confidentiality. The names and contact details of attendees indicated in the minutes of a stakeholders’ meeting were not revealed.

## Trustworthiness

The trustworthiness of the study was enhanced by introducing strategies as suggested by Lincoln and Guba (in Botma et al. 2010), including prolonged engagement and triangulation to enhance credibility, thick description to enhance transferability and dependability, and the availability of an auditable trail to enhance confirmability.

## Discussion

### Outline of the results

The development of the contemporary curriculum took place over two years. The macro curriculum was determined by the regulatory bodies for nursing (South African Nursing Council) and education (Council on Higher Education).

Nursing qualifications are regulated by the South African Nursing Council (South Africa 2005), including specialisation in advanced midwifery. The South African Nursing Council (South Africa 2005) prescribes the minimum requirements and competencies of advanced midwives which have to be reflected in the curriculum.

The Council on Higher Education prescribes the classification of all formal qualifications in South Africa according to levels (levels 1–10) on the Higher Education Qualification Sub-Framework (Council on Higher Education 2014) with level descriptors to define the critical cross-field outcomes and associated exit level outcomes of each level. It further necessitates the adoption of outcome-based education as the basic philosophy of education (South Africa 1997; 2008).

The module codes and descriptions, credits, entry and admission requirements and the duration of the programme remained unchanged according to the educational institution’s regulations, as approved by Senate (University of X 2012).

The meso-level framework addressed the content of the modules and outcomes related to foundational competence (knowledge), practical competence and reflexive competence. The meso-level framework was further developed during a workshop with stakeholders from practice, education and policy-making authorities (University of X 2011). The stakeholders were important sources of information and should be involved in future collaboration (Keogh et al. 2010). Their contribution was significant regarding needs, priorities and challenges from their respective fields. The content and outcomes were further refined with inclusion of information from national and international authoritative and research documents. Needs that were highlighted included staff shortages, population growth, changing health profiles and global trends.

Health care workers, including nurses and midwives, migrate abroad to work (Oosthuizen & Ehlers 2007) contributing to a shortage of nurses and midwives in South Africa. Remaining nurses and midwives are overworked and hospitals are understaffed (Alliance of African Midwives 2011). As a consequence, there is an increased demand for competent midwifery leaders. Continuous population growth fuels the demand for more midwives to be trained at an advanced level. Advanced midwives can provide holistic health care, particularly in primary health care clinics where medical doctors are often absent (Alliance of African Midwives 2011).

The South African National Department of Health (2013) aims to address changing health care needs by identifying national health priorities and implementing various strategies. Maternal and child health was identified as one of the top five priorities. Important strategies to reduce maternal and child mortality and morbidity include a primary health care approach to enhance provision of early and quality ante- and postnatal services; health worker initiated counselling and testing for HIV and prevention of mother-to-child transmission as part of antenatal care; review and strengthening of referral systems for pregnant women, newborns and children with high-risk conditions; training and enforcement of infection control measures in all maternity and neonatal facilities; introducing community health care workers to conduct postnatal care home visits to identify problems with the mother and her baby; and establishing units for contraception and termination of pregnancy at all facilities (Department of Health, S.A. 2013). In the interest of the country, it was important to take note of these strategies when revising the curriculum for advanced midwifery.

The new curriculum was informed by globalisation, while being locally relevant. For instance, the educational philosophy underpinning the master’s programme in England links interprofessional learning closely to work-based learning and assessment to improve collaborative linking with medical colleagues, raising of trainee profiles and acceptance of an extended role in providing safe and quality care to women in pregnancy and childbirth (Gaskell & Beaton 2015).

### Practical implications

The improved contemporary curriculum was converted to a micro-curriculum, with inclusion of study guides, a broad range of supplementary materials, instructional exercises for particular units and additional available resources to achieve the desired outcomes. Teaching and learning strategies were revised to enhance student-centred learning using technology and social media. Midwifery and educational experts were requested to review the contemporary curriculum and final refinement was done. The curriculum was ready for the next phase of the study, namely diffusion into the education system.

### Limitations of the study

The study was limited to a single setting, of which only the first two steps were described.

### Recommendations

The process followed for development of a contemporary curriculum paved the way for implementation and external benchmarking, which will be addressed in a further article. The process used to get to the contemporary curriculum can be repeated by other institutions for different professional specialities.

## Conclusion

The development of a contemporary curriculum was a journey worth exploring which contributed significantly to an improved curriculum for advanced midwifery. Curriculum development is a dynamic process which should continuously realign with national health care priorities and international trends in education.
